# Repeatability of vascular responsiveness measures derived from near‐infrared spectroscopy

**DOI:** 10.14814/phy2.12772

**Published:** 2016-05-04

**Authors:** Kaitlin M. McLay, Josh P. Nederveen, Silvia Pogliaghi, Donald H. Paterson, Juan M. Murias

**Affiliations:** ^1^ Canadian Centre for Activity and AgingThe University of Western OntarioLondonOntarioCanada; ^2^ School of KinesiologyThe University of Western OntarioLondonOntarioCanada; ^3^ Department of Neurological and Movement SciencesUniversity of VeronaVeronaItaly; ^4^ Faculty of KinesiologyUniversity of CalgaryCalgaryAlbertaCanada

**Keywords:** Day‐to‐day reliability, endothelial function, oxygen saturation, reactive hyperemia, test‐to‐test reliability, vasodilation

## Abstract

Near‐infrared spectroscopy (NIRS)‐derived measures of tissue oxygen saturation (StO_2_) have been recently shown to significantly correlate with the widely used method for noninvasively assessing vascular endothelial function, flow‐mediated dilation (FMD). The purpose of this study was to examine the intraday and interday reliability of the reperfusion slope of StO_2_ (slope 2 StO_2_) and compare it to FMD. Ultrasound‐derived FMD was quantified following 5 min of distal cuff occlusion of the popliteal artery in nine healthy young men (26 ± 3 years). An FMD test was performed each of 4 days, with a fifth involving three tests. FMD was calculated as the greatest percent change in diameter from baseline (%FMD). StO_2_ was measured using NIRS throughout each test, with slope 2 StO_2_ being calculated as the upslope of 10‐sec following cuff release. Reliability was determined using repeatability, intraclass correlation coefficients (ICC), and coefficient of variation (CV). Repeatability of slope 2 StO_2_ was better than %FMD for both intraday (0.43 and 5.65, respectively) and interday (0.48 and 4.82, respectively) comparisons; approximately 30% of mean values for slope 2 StO_2_ could be attributed to measurement error, whereas 100% of mean FMD could be for both intraday and interday comparisons. Similarly, ICC and CV values indicated stronger reliability of slope 2 StO_2_ compared to %FMD for both intraday (ICC 0.92 and 0.36, respectively; CV 9 ± 4% and 44 ± 24%, respectively) and interday (ICC 0.94 and 0.25, respectively; CV 14 ± 5% and 40 ± 22%, respectively) comparisons. In conclusion, NIRS‐derived slope 2 StO_2_ can be used as a reliable measure of vascular reactivity.

## Introduction

Impaired endothelial‐dependent vasodilation is an important feature of vascular disease and is strongly associated with several chronic cardiovascular conditions (Neunteufl et al. 2000; Kuvin et al. 2001; Perticone et al. 2001; Gokce et al. 2002; Modena et al. 2002; Widlansky et al. 2003). The reactive hyperemia endothelial function test, commonly referred to as a flow‐mediated dilation (FMD) test, is a widely used, noninvasive technique which provides insight into peripheral conduit artery vasoreactivity and information about the integrity and function of the endothelium (Vita and Keaney 2002).

The FMD technique, typically assessed in peripheral conduit arteries such as the brachial (Betik et al. 2004), radial (Brook et al. 2005), superficial femoral (Kooijman et al. 2008), and popliteal (Green et al. 2010), has increasingly been applied in both clinical and physiological studies. Although FMD is an important tool in assessing vascular and endothelial function, there is some concern regarding the reliability of the measurement. Some studies have reported that the test‐retest repeatability of %FMD, as measured by the coefficient of variation (CV) statistic, can be markedly worse than that of the baseline and peak diameters (Herrington et al. 2001). Additionally, other studies have shown that %FMD may be a satisfactory, or a very poor (Brook et al. 2005; Peretz et al. 2007), indicator of vascular function due to high variability between repeated measures.

A new approach to assess vascular responsiveness has emerged with the use of near‐infrared spectroscopy (NIRS). Recently, tissue oxygen saturation (StO_2_) was measured distal to the occlusion site during a FMD test and the NIRS‐derived reperfusion slope immediately following ischemia (slope 2 StO_2_) was indicated to be a good measure of vascular responsiveness (McLay et al. 2016), which could be used to test responses to various interventions (i.e., exercise training, diet or pharmacological). Additionally, this new approach to assess vascular function can be easily employed in the leg, something that is more difficult with FMD due to the small diameter changes relative to the large vessel diameter. This is an important feature as being able to evaluate changes in vascular responsiveness in the regions where those changes are often expected to occur (e.g., in the lower limbs before and after an exercise training intervention or chronic adaptations to exercise) might contribute to a better characterization of this response. Furthermore, measurements of StO_2_ have been previously conducted in clinical settings and have reported results that suggest that NIRS‐derived measures, specifically the reperfusion rate, were able to monitor differences in hemodynamic responses. Creteur et al. (2007) found that the reperfusion slope was higher in patients with severe sepsis who survived than in nonsurvivors. Additionally, they reported that the reperfusion slope tended to increase in survivors over the observation period but not in nonsurvivors.

With the increasing use of noninvasive techniques to assess vascular responsiveness both in clinical settings and in physiological research, it is important to have a better understanding of the reliability of measures being used. To our knowledge, no study to date has systematically evaluated the day‐to‐day and test‐to‐test reliability of the recently proposed measurement of vascular reactivity (slope 2 StO_2_) in the leg, or compared the reliability to that of %FMD in the popliteal artery, which is a necessary step if this new approach is to be applied to compare responses before and after an intervention or simply at different time points. Therefore, the main purpose of this study was to examine the test‐to‐test reliability (variability between repeated tests within a single day) and day‐to‐day reliability of the NIRS‐derived measure slope 2 StO_2_, and compare it to the widely used FMD measurement.

## Methods

### Participants

Nine healthy young men (mean ± SD, age: 26 ± 3 years; mass: 82 ± 8 kg; height: 178 ± 4 cm) volunteered and gave written consent to participate in the study. All procedures were approved by The University of Western Ontario Research Ethics Board for Health Sciences Research Involving Human Subjects. All participants were recreationally active (i.e., meeting the recommended guidelines for physical activity (Tremblay et al. 2011)) and nonsmokers. Additionally, all subjects were normotensive (mean blood pressure ± SD: systolic 124 ± 7 mmHg; diastolic 66 ± 7 mmHg) and no subjects were taking medications that would affect hemodynamic responses.

### Study design

A series of FMD tests were performed on each participant over five consecutive days, with NIRS‐derived measures of StO_2_ obtained throughout the duration of each test. All tests were performed in an environment where temperature was controlled throughout the testing protocol (20–22°C) and at the same time each day to minimize diurnal effects. All participants were instructed to refrain from caffeine, alcohol, and exercise for >12 h prior to their scheduled visit. One FMD test was performed on each of 4 days, with a fifth day involving three FMD tests. The day when three FMD tests were performed was randomized between subjects and each of the three FMD tests were separated by a 30‐min rest period to allow blood flow and arterial dilation to return to resting conditions (Harris et al. 2006). Each FMD test was performed with an occlusion pressure of 250 mmHg.

### Near‐infrared spectroscopy

StO_2_ of the tibialis anterior muscle was monitored continuously throughout each FMD test with a frequency‐domain multidistance NIRS system (Oxiplex TS, ISS, Champaign, IL). Briefly, the system was composed of a single channel consisting of eight laser diodes operating at 2 wavelengths (*λ *= 690 and 828 nm, 4 at each wavelength), which were pulsed in rapid succession, and a photomultiplier tube. The lightweight plastic NIRS probe (connected to laser diodes and a photomultiplier tube by optical fibers) consisted of two parallel rows of light emitter fibers and one detector fiber bundle; the source‐detector separations for this probe were 2.0, 2.5, 3.0, and 3.5 cm for both wavelengths. The probe was placed on the belly of tibialis anterior the muscle (midway between the knee and the ankle), was secured in place with an elastic strap tightened to prevent movement and was covered with an optically dense, black vinyl sheet, thus minimizing the intrusion of extraneous light. An elastic bandage was applied to further minimize intrusion of extraneous light and probe movement. A pneumatic cuff (Adult 11 long, Flexiport; Welch Allyn Inc., Skaneateles Falls, NY) was placed just below the knee, above but not over the secured NIRS probe. By measuring changes in light absorption at different wavelengths, changes in oxyhemoglobin (HbO_2_) and deoxyhemoglobin (HHb) can be measured continuously, and StO_2_ can be calculated (defined as [HbO_2_]/[HbO_2_ + HHb]). NIRS measurements were collected continuously for the entire duration of each FMD test (2‐min baseline, 5‐min occlusion, and 5‐min postrelease) plus an additional 3 min following cuff release to ensure StO_2_ fully returned to baseline levels (for a total of 8 min postrelease).

The NIRS device was calibrated at the beginning of the first test session following an instrument warm‐up period of at least 20 min. The calibration was done with the probe placed on a calibration block (phantom) with absorption (*μ*_a_) and reduced scattering coefficients (*μ*_s’_) previously measured; thus, correction factors were determined and were automatically implemented by the manufacturer's software for the calculation of the *μ*_a_ and *μ*_s’_ for each wavelength during the data collection. Calculation of [HbO_2_] and [HHb] reflected continuous measurements of *μ*_s’_ made throughout each testing session (i.e., constant scattering value not assumed). The probe remained secured to the leg throughout the duration of the visit to ensure measurement consistency between both FMD tests. Data were stored online at an output frequency of 2 Hz, but were reduced to 1 s bins for all subsequent analyses within this study.

Baseline StO_2_ (%) was calculated as the average of 1 min of StO_2_ prior to ischemia. Minimum StO_2_ (%) was calculated as the lowest StO_2_ value attained during ischemia. The StO_2_ reperfusion rate was quantified as the upslope of a 10 sec window immediately following cuff release of the StO_2_ signal (slope 2 StO_2_, %/s); the reperfusion rate immediately following cuff release is a relatively linear response which allows for a simple slope calculation. Peak StO_2_ (%) was calculated as the highest StO_2_ value reached following cuff release.

### Popliteal artery assessments

Flow‐mediated dilation of the popliteal artery was assessed in accordance with previously published guidelines for the current standardized methodology (Corretti et al. 2002; Thijssen et al. 2011). Following at least 10 min of supine rest, participants were instructed to lie prone as ultrasound imaging was performed on the back of the knee. A small pillow was placed under the participant's ankle for comfort and optimization of the knee angle so there was no leg movement throughout the cycles of the FMD tests. The left popliteal artery was imaged immediately proximal to the bifurcation (usually at or slightly above the popliteal fossa), and a pneumatic cuff (Flexiport; Welch Allyn Inc.) was placed around the calf (approximately 5 cm distal to the popliteal fossa). Heart rate was continuously monitored with a three‐lead ECG to allow for consistent and accurate selection of arterial diameter measurements at the end of the diastolic phase of the cardiac cycle.

The popliteal artery was imaged with a 10‐MHz multifrequency linear‐array transducer attached to a Doppler ultrasound machine (VingMed System FiVe, GE Medical Systems, Horten, Norway). All scans were performed by an experienced investigator. All scans were made with similar ultrasound settings and all images were recorded on an external video camera (HDD Everio; JVC, Mississauga, ON, Canada) for later offline analysis. Baseline diameter was recorded prior to manual inflation of the pneumatic cuff. The cuff was then inflated for 5 min to an occlusion pressure of 250 mmHg, during which diameter was not recorded. Fifteen seconds prior to release of the cuff the video camera resumed recording and at exactly 5 min after inflation, the pneumatic cuff was released and arterial diameter was continuously monitored for 5 min post release.

Diameter measurements, defined as the distance between the media and intima interface of the near wall and far wall, were obtained using a caliper that converted image pixels to millimeters. Triplicate measurements of diameter were taken for each of five baseline images and averaged to determine the baseline diameter of the artery. Similarly, triplicate measurements of diameter were averaged for images taken every 15 sec following cuff release. Peak diameter was determined as the postocclusion image with the largest diameter and percent flow‐mediated dilation (%FMD) was then calculated as the percent change in diameter from baseline.

### Statistical analysis

All statistical analyses were performed using SPSS software, version 19 (SPSS Inc., Chicago, IL) and Microsoft Excel 2010 (Microsoft, Seattle, WA).

Group mean, standard deviation (SD) and coefficient of variation (CV = SD/mean × 100) were calculated for NIRS‐ and ultrasound‐derived parameters for each test. A one‐way repeated measures analysis of variance (ANOVA) was used to determine if there were significant differences within the variables of the five NIRS and FMD tests performed over consecutive days, and the three NIRS and FMD tests performed within the same day. The repeatability, also known as the coefficient of repeatability, of each variable for the comparisons was calculated by multiplying the within‐subject standard deviation (Sw) by 2.77 [or (1.96 × √2) × Sw] (Bland and Altman 1996). The repeatability represents the critical value at which a measurable change is observed in a given participant between tests. Reliability of three FMD tests repeated in a single day and the five tests performed over consecutive days were assessed using the intraclass correlation coefficient (ICC_(1,1)_), which was based on the repeated measures ANOVA with testing session as the independent variable (Shrout and Fleiss 1979). For statistical tests *P* < 0.05 was considered significant.

## Results

### Near‐infrared spectroscopy

**Figure 1:**
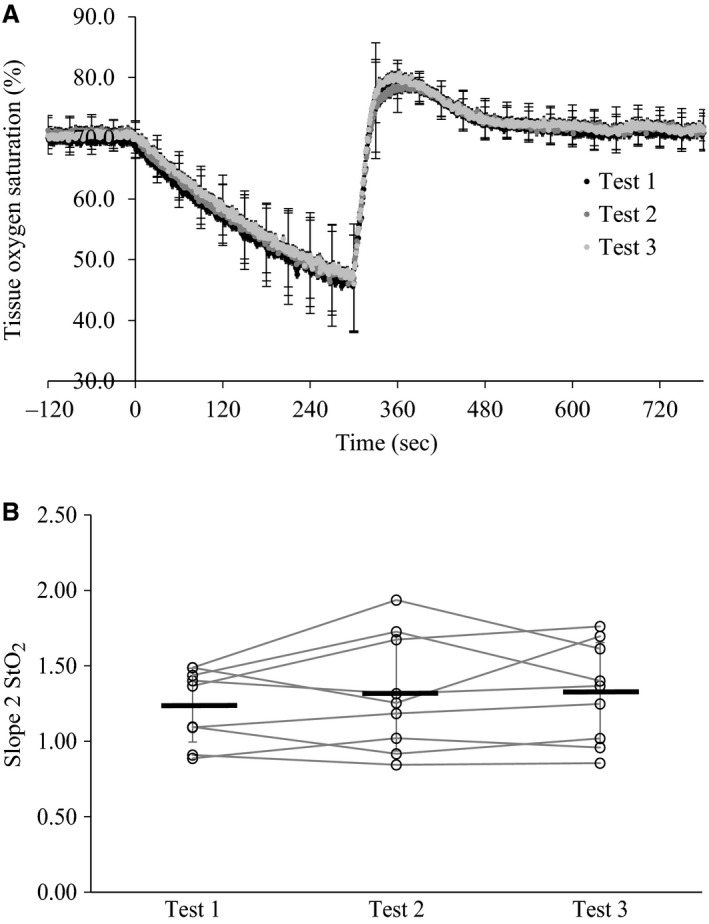
(A) Group average tissue oxygen saturation profile for three vascular occlusion tests performed within the same day; (B) Group mean values for slope 2 StO_2_ each day are represented by the thick black lines, with each open circle and connecting line representing an individual subject.

**Figure 2:**
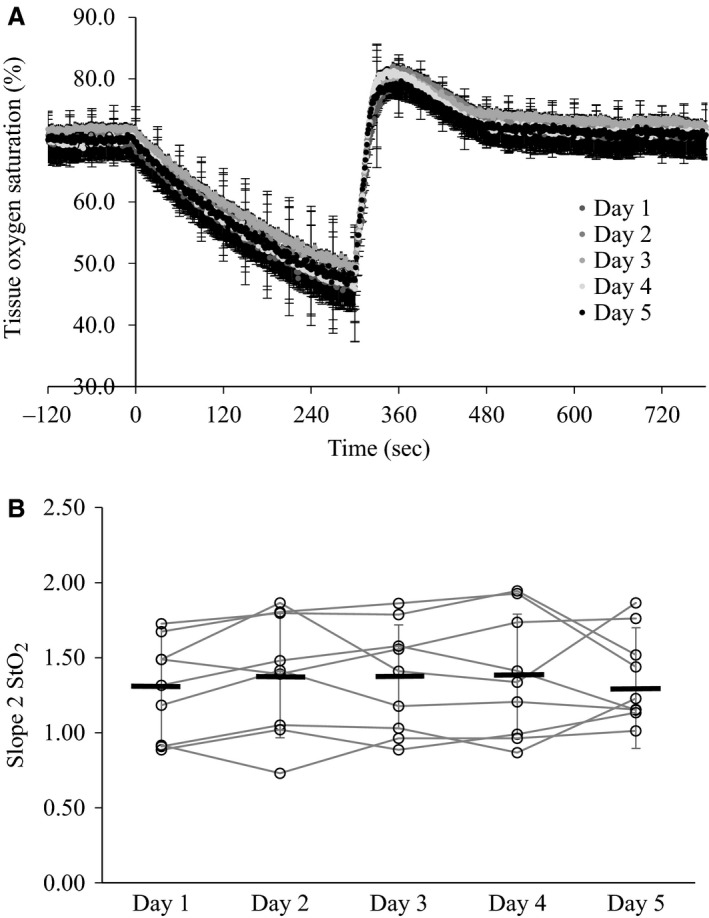
(A) Group average tissue oxygen saturation profile for each of five vascular occlusion tests performed on separate days; (B) Group mean values for slope 2 StO_2_ each day are represented by the thick black lines, with each open circle and connecting line representing an individual subject.

Group means and standard deviation for StO_2_ parameters for within and between‐day comparisons are listed in Tables 1 and 2, respectively. There was no significant difference in slope 2 StO_2_ between tests performed within a single day, or across 5 days. Repeatability values for the intraday and interday comparisons were 0.43 and 0.48, respectively; which represents that 33% and 36% of the mean value could be attributed to measurement error. ICC for the intraday and interday comparisons were 0.92 and 0.94, respectively. CV for the intraday and interday comparisons were 9 ± 4% (range 3–15%) and 14 ± 5 (range 9–24). Figure 1 shows the average profile for each of three tests performed in a single day (Panel A), as well as the variation in slope 2 StO_2_ values for each individual and group means (Panel B). Figure 2 shows the average profile for each of the five tests performed over 5 days (Panel A), as well as the variation in slope 2 StO_2_ values for each individual and group means (Panel B).

**Table 1:** Ultrasound‐ and NIRS‐derived measurements throughout a vascular occlusion test for three tests performed in a single day

	Test 1	Test 2	Test 3
Ultrasound‐derived measures			
Baseline diameter (mm)	6.7 ± 0.5	6.5 ± 0.7	6.6 ± 0.8
Peak diameter (mm)	6.9 ± 0.6	6.8 ± 0.7	6.9 ± 0.8
Flow‐mediated dilation (%)	3.1 ± 2.0	4.5 ± 2.5	5.6 ± 3.5
NIRS‐derived measures			
Baseline StO_2_ (%)	71.1 ± 2.4	69.6 ± 1.7	70.7 ± 2.9
Absolute Minimum StO_2_ (%)	46.2 ± 7.5	46.0 ± 6.6	45.9 ± 7.5
Absolute Peak StO_2_ (%)	82.1 ± 1.4	81.5 ± 1.8	82.2 ± 2.0
Slope 2 StO_2_ (%/s)	1.32 ± 0.38	1.24 ± 0.25	1.32 ± 0.33

Values are means ± standard deviations. NIRS, near‐infrared spectroscopy.

**Table 2:** Ultrasound‐ and NIRS‐derived measurements throughout a vascular occlusion test for five tests performed across 5 days

	Day 1	Day 2	Day 3	Day 4	Day 5
Ultrasound‐derived measures					
Baseline diameter (mm)	6.7 ± 0.7	6.7 ± 0.5	6.7 ± 0.6	6.5 ± 0.5	6.7 ± 0.5
Peak diameter (mm)	6.9 ± 0.6	6.9 ± 0.6	7.0 ± 0.6	6.8 ± 0.5	7.0 ± 0.4
Flow‐mediated dilation (%)	3.1 ± 2.0	3.5 ± 1.6	4.8 ± 1.8	4.5 ± 2.3	4.5 ± 2.0
NIRS‐derived measures					
Baseline StO_2_ (%)	71.1 ± 2.4	71.7 ± 3.6	72.3 ± 2.6	70.4 ± 4.0	70.4 ± 3.5
Absolute minimum StO_2_ (%)	46.2 ± 7.5	46.6 ± 9.9	49.2 ± 5.9	46.6 ± 8.0	46.1 ± 7.7
Absolute peak StO_2_ (%)	82.1 ± 1.4	83.4 ± 2.7	82.7 ± 1.7	82.8 ± 2.5	81.5 ± 4.5
Slope 2 StO_2_ (%/s)	1.32 ± 0.38	1.38 ± 0.41	1.37 ± 0.35	1.39 ± 0.40	1.30 ± 0.40

Values are mean ± standard deviations. NIRS, near‐infrared spectroscopy.

### Flow‐mediated dilation

**Figure 3:**
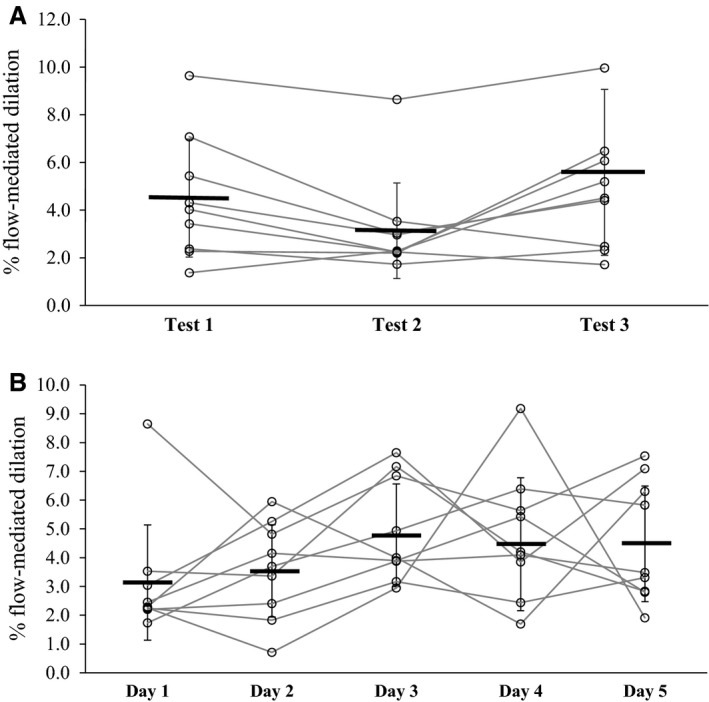
(A) Group mean values for each of three FMD tests performed in a single day are represented by the thick black lines, with each open circle and gray line representing an individual subject; (B) Group mean values and individual data for each of five FMD tests performed across 5 days.

Group means and standard deviation for baseline diameter, peak diameter, and %FMD for within and between‐day comparisons are listed in Tables 1 and 2, respectively. There was no significant difference for %FMD between tests performed within a single day, or across 5 days. Repeatability values for the intraday and interday comparisons were 5.62 and 4.82, respectively; which means that greater than 100% of the mean value for FMD could be attributed to measurement error both within and between days. ICC for the intraday and interday comparisons were 0.36 and 0.25, respectively. CV for the intraday and interday comparisons were 44 ± 24% (range 7–79%) and 40 ± 22% (range 3–88%). Figure 3 shows the variation in %FMD values for each individual, as well as group means, for three tests within a single day (Panel A) and five tests across 5 days (Panel B).

## Discussion

The main goal of this study was to investigate the test‐to‐test (intraday) and day‐to‐day (interday) reliability of the NIRS‐derived slope 2 StO_2_ and compare it to that obtained from ultrasound‐derived %FMD of the popliteal artery. The main findings were as follows: (1) the reliability of the slope 2 StO_2_ measure was strong both within and between testing days (low repeatability values, high ICCs, and low CV); (2) the reliability of %FMD, at least in the artery measured in this study, was poorer than that observed for the StO_2_, both within and between testing days, as indicated by high repeatability values, low ICCs, and high CV.

Although NIRS‐derived measures of StO_2_ have been shown to reflect vascular responsiveness (McLay et al. 2016), and they have been used to measure StO_2_ in healthy and clinical populations previously (Creteur et al. 2007; Doerschug et al. 2007), this study was the first to comprehensively examine the reliability of the NIRS‐derived parameter slope 2 StO_2_ in the leg. This is important as repeatability is used to examine the influence of measurement errors on data analysis and is an indicator of absolute reliability (Bland and Altman 1996), which is necessary to know when interpreting changes in response to an intervention or as a consequence of factors such as training status, disease, etc. In this study, small values for repeatability indicated strong reliability of the measures. Repeatability values were indicative of strong reliability of the measure for both intraday (0.43) and interday (0.48) comparisons, with approximately 30% of the measurement potentially being influenced by measurement error. This study also reported the ICC, which provides a measure of relative reliability. As suggested by Portney and Watkins (Portney and Watkins 2000), ICC values >0.75 are considered to be reliable. In this study, ICC values for intraday (0.92) and interday (0.94) reliability of slope 2 StO_2_ indicated very strong reliability of the technique. Similarly, CV was indicative of strong reliability of the measure both within (9 ± 4%) and between days (14 ± 5%). Comparable CV values for slope 2 StO_2_ in the leg were previously reported by McLay et al. (2016); however, of note, in that study the CV was derived from only two tests performed in a single day. This study examined the reliability not only within a single day (with three repeats) but also across multiple days, something of particular value for intervention‐type studies. These results are consistent with a previous study that reported the CV of StO_2_ in combination with vascular occlusion in the thenar eminence of healthy controls. Gómez et al. (2008) reported high reliability of StO_2_ for three occlusion tests performed within the same day (CV 14.2 ± 9.2%). This study examined the reliability of the slope 2 StO_2_ in a more systematic way than Gómez et al. and also reported strong day‐to‐day reliability for tests performed on five consecutive days.

In this study, slope 2 StO_2_, a relatively new approach to assessing vascular responsiveness which has been shown to significantly correlate to %FMD (McLay et al. 2016), showed better reliability than that of the widely used measure of FMD. Repeatability values were high for intraday and interday FMD such that a difference of 5.6% and 4.8%, respectively, would be needed to observe a change in FMD that would not be associated with measurement error of the technique. These values for repeatability are as large as the mean FMD measures themselves. Similarly, ICC values for both intraday (0.36) and interday (0.25) comparisons were indicative of poor reliability. It has to be acknowledged that this study utilized the popliteal artery for FMD measures, which could result in poorer reliability as compared to what might be observed in arteries that are more commonly assessed, such as the brachial or the radial artery. However, it has to be noted that poor reliability has been reported in other measurement sites as well. Hardie et al. (1997) demonstrated that reproducibility of brachial artery FMD was poor and likely to provide inaccurate measurements for two FMD tests separated by an average of 90 days. Repeatability calculated from reported values for within subject SD indicated that changes in FMD in the brachial artery would need to be approximately 19% to be able to detect differences that could not be attributed to measurement error. Similarly, Brook et al. (2005) assessed intra‐ and interday reliability for two FMD tests performed in the same day and two tests performed approximately 7 days apart. The repeatability values were high for both intraday (11%) and interday (11%). Thus, even though the relevance of the %FMD as a measure of vascular responsiveness is undeniable, this lack of reliability that is often acknowledged in the literature and that it is also supported by this current experimental dataset, might be one of the factors contributing to the lack of differences in %FMD between groups that were expected to show different responses (Atkinson and Batterham 2013; Birk et al. 2013). A measure such as the slope 2 StO_2_ can contribute to the study of noninvasive assessment of vascular responsiveness not only by providing another technique that can estimate vascular reactivity within the microvasculature, but also by offering the possibility of detecting between group differences more easily (i.e., needing a lower number of participants) thanks to the greater reliability of this measure.

The measurement of StO_2_ throughout a vascular occlusion has been previously used in clinical settings to monitor recovery of patients. Studies have shown significant differences between the reperfusion rate of the StO_2_ signal (slope 2 StO_2_) of septic patients and healthy controls, with the septic patients having a much slower reperfusion following cuff release than that of controls (Doerschug et al. 2007). Additionally, repeated measurements of slope 2 StO_2_ in intensive care patients has shown that slopes increase over time in surviving patients, but not in nonsurvivors (Doerschug et al. 2007). These studies, in combination with recent demonstration of the association between slope 2 StO_2_ and %FMD (McLay et al. 2016), demonstrate the ability of this measure to detect differences in vascular and hemodynamic responses.

Vascular impairments at both the macro‐ and the microcirculatory level are known to be associated to cardiovascular disease (CVD) (Lloyd‐Jones et al. 2010). As such, proper assessment of vascular responsiveness becomes an important instrument for early detection of preclinical dysfunction, diagnosis, monitoring of treatment efficacy and possibly prevention of CVD. The use of NIRS allows for changes in microvascular reactivity to be assessed at the level of the muscle instead of solely relying on conduit artery estimates of vascular reactivity. This is an important feature as some forms of CVD have been indicated to originate with functional limitations within the microcirculation (Seals 2014). Thus, detecting problems where they originate may help early detection of future cardiovascular complications. In this study, assessing vascular responsiveness in the leg was an important factor when selecting the area of FMD and NIRS interrogation as several training intervention are predominately lower limb exercises (such as cycling and running) and thus, even though it is acknowledged that changes in vascular reactivity can extend to other areas, it is important to be able to assess vascular responsiveness in the region that is more affected by the proposed intervention. Nonetheless, with any measurement technique it is important to understand the degree to which differences in measures may be attributed to physiological adaptations to various interventions or clinically meaningful changes instead of reflecting measurement error. The strong reliability of this new technique to measure vascular responsiveness, taken together with the previously established capability of tracking changes in hemodynamic responses, makes it a promising application for assessing vascular responsiveness as an index of endothelial health, and in monitoring responses to various interventions.

A criticism of this study could be that %FMD was calculated from diameter measurements made every 15 sec, as the most recent recommendations for the analysis of FMD stipulate the use of edge‐tracking software for the assessment of arterial diameters for each cardiac cycle in the postocclusive period. A previous analysis comparing manual and edge‐tracking software (data not presented) found that there was no difference between FMD values obtained through the two analysis methods. Additionally, although the automated edge‐tracking software does include more diameters for the determination of %FMD, there is more noise in the data as a result of the variability in the greater number of diameters which results in poorer reliability of the measure derived from the edge‐tracking software. In this context, the values reported here are derived from the less variable %FMD of the manual analysis to avoid exacerbating the low repeatability of the FMD measure. This study did not focus on the reliability of the FMD measure but instead emphasizes the strong reliability of the new approach to assessing vascular reactivity (i.e., NIRS‐derived measures of slope 2 StO_2_).

This study also reports values for baseline StO_2_, Min StO_2_, and Peak StO_2_ as a reference for future studies. It should be noted that the static values for StO_2_ are not “corrected” for adipose tissue thickness (ATT), which has been suggested to influence the measurement of these values (Niwayama et al. 2000). Nevertheless, while ATT may influence the absolute values of StO_2_ obtained for certain parameters it has been reported that dynamic changes, such as slope 2 StO_2_, should not be affected as the changes are independent of absolute values (Bopp et al. 2011). That being said, the TA muscle offers an ideal sight of measurement for NIRS as there is often less adipose tissue overlying the muscle compared to other muscles in the leg.

In conclusion, this study demonstrated that the NIRS‐derived slope 2 StO_2_, a measure established to reflect vascular reactivity (Creteur et al. 2007; Doerschug et al. 2007; McLay et al. 2016), has strong reliability. The reliability of this new approach for noninvasively assessing vascular responsiveness has important implications for the assessment of vascular responsiveness as it might contribute in determining differences between groups or before and after an intervention that would be otherwise difficult to establish.

## Conflict of Interest

No conflicts of interest, financial or otherwise, are declared by the author(s).
